# *“We were being treated like the Queen”*: understanding trial factors influencing high paediatric malaria treatment adherence in western Kenya

**DOI:** 10.1186/s12936-017-2164-6

**Published:** 2018-01-05

**Authors:** Caroline Jones, Ambrose O. Talisuna, Robert W. Snow, Dejan Zurovac

**Affiliations:** 1 KEMRI-Welcome Trust Research Programme, Nairobi, Kenya; 2 Centre for Tropical Medicine and Global Health, University of Oxford, Oxford, UK

**Keywords:** Adherence, Artemether–lumefantrine, SMS, Trial factors, Quality of care

## Abstract

**Background:**

Adherence to anti-malarial medication is highly variable but frequently suboptimal. Numerous interventions with a variety of methodological approaches have been implemented to address the problem. A recently conducted, randomized, controlled trial in western Kenya evaluated the effects of short message service (SMS) reminders on paediatric adherence to artemether–lumefantrine (AL) and found over 97% adherence rates in both intervention and control arms. The current study was undertaken to explore participants’ experiences in the trial and identify the factors contributing to the high adherence rates.

**Methods:**

In July 2016, 5 months after the trial completion, focus group discussions (FGDs) were undertaken with caregivers of children who had been treated in the intervention (n = 2) or control (n = 2) arms and who, post-trial, had received malaria treatment from the same facilities. The FGDs explored similarities and differences in perceptions and experiences of the care they received during and after the trial.

**Results:**

Intervention-arm participants reported that SMS messages were effective dosing reminders. Participants from both arms reported that trial instructions to keep empty AL packs for verification during a home visit by a health worker affected their dosing and adherence practices. Differences between trial and post-trial treatment experiences included: administration of the first AL dose by health workers with demonstration of dispersible tablets dilution; advice on what to do if a child vomited; clear instructions on timing of dosing with efforts made to ensure understanding; and, information that dose completion was necessary with explanation provided. Participants reported that after the trial AL was not available at facilities, constraining their ability to adhere to recommended malaria treatment. They emphasized receiving respectful and personal treatment from trial health workers contributing to perceptions of high quality care and enhanced readiness to adhere to dosing instructions.

**Conclusions:**

This study highlights the complex range of factors that influence AL adherence. The results suggest that in addition to standardized definitions and measurement of adherence, and the influence of enrolment procedures, AL adherence trials need to take account of how intervention impact can be influenced by differences in the quality of care received under trial and routine conditions.

## Background

Poor patient adherence to anti-malarial medication increases the risk of treatment failure and contributes to the development and spread of anti-malarial drug resistance [1–3]. Numerous studies measuring adherence to artemether–lumefantrine (AL), the most commonly used artemisinin-based combination therapy (ACT) for malaria in Africa, have frequently reported suboptimal results and wide variability of adherence levels, ranging from 39 to 100% [4, 5]. Similarly, reviews of studies examining factors influencing adherence have not shown consistent patterns in the determinants of adherence [4, 5] nor have concluded about the most effective interventions to improve adherence to anti-malarial drugs [6]. Methodological differences with respect to adherence definitions, measurement methods, study designs, different anti-malarial medicines evaluated, and diversity of context in which adherence studies were undertaken, contribute to the lack of consistency and conclusive evidence. Despite these methodological challenges, some reviewers [6] have suggested that a package of interventions (e.g., patients’ visual and verbal information with community education) should be promoted to improve adherence to anti-malarial treatment while alternative interventions, such as short message service (SMS) reminders, which have been shown to be effective for chronic diseases [7, 8], deserve further rigorous evaluation of their effectiveness for enhancing adherence to malaria treatment.

In Siaya county in western Kenya, an area of high malaria risk, a randomized controlled trial was conducted to determine the efficacy of SMS mobile phone reminders on adherence to AL therapy in children under 5 years of age with uncomplicated malaria. Caregivers of children with confirmed malaria were randomized at four study health facilities to either the intervention (i.e., to receive SMS reminders to administer AL plus standard care) or the control arm (i.e., to receive only standard care). The automated SMS reminders were sent 8 h after the first AL dose and then every morning (08.00) and evening (20.00) until the full AL course of 6 doses was administered. The content of message was as follows:

*Hello [name of care giver], have you remembered to give your child the [dose number] dose of malaria medicine? If not, please do so. Thank you, [Name of HF].*

In both arms, all children treated with AL received care by study personnel in line with treatment, dispensing and counselling standards specified in the national guidelines [9]. Detailed descriptions of the pre-trial feasibility assessment, SMS intervention, standard care and the design and findings of the trial have been elaborated in the previous publications [10–13]. In summary, the key finding of the trial was very high adherence to AL in both arms, with 97.8% of patients completing all doses in the intervention and 97.6% in the control arm. The post-trial phase, reported in this paper, involved an exploratory qualitative study to understand the influences contributing to the observed trial outcomes. In light of the very high adherence rates recorded in the trial, the specific objectives of the study were to explore the experiences of the trial participation among caregivers and to identify the factors contributing to the high adherence rates observed in both arms.

## Methods

This exploratory qualitative study used focus group discussions (FGDs) with caregivers who had participated in the trial to explore experiences of receiving and administering malaria treatment throughout the trial and during a post-trial period. Data collection was undertaken in July 2016, 5 months after completion of the trial, in order to allow sufficient time for completion of the data analysis determining the effects of the intervention. Given the high level of adherence in both arms of the trial, the focus of the post-trial qualitative research was on assessing potential ‘trial effects’ through an exploration of the experiences of malaria diagnosis and treatment at the health facility among caregivers during the trial, compared with their experiences in the post-trial period. Participants from both the intervention and control arm, who were identified as being ‘adherent’ during the trial and who in the post-trial period had a child treated for malaria, were purposefully selected to take part in FGDs to describe their experiences. In the group discussions, the caregivers were asked to compare their experiences and medicine administration practices during the trial with a similar scenario when they had a child aged under 5 years diagnosed with malaria at the same health facilities under routine conditions after the trial.

Adherent caregivers who were recruited within the last 3 months of the trial were identified from the trial database. These potential participants were contacted individually by phone to determine if they had visited one of the health facilities since the end of the trial, seeking treatment for a child aged under 5 years who was subsequently diagnosed with malaria. Out of 85 potentially eligible caregivers identified, 41 met the inclusion criteria and were invited to attend a FGD on the effects of the trial. The topic guide was developed around four areas: (1) recap of the trial and its related activities; (2) caregiver experiences and medicine administration practices during the trial; (3) post-trial experiences and medicine administration practices; and, (4) comparison of the trial and post-trial experiences and practices. The FGDs were held at Bondo Hospital, one of the trial sites and moderated by an experienced community health worker who had also guided group discussions during the intervention development phase of the study. All groups were conducted in *Dholuo*, the caregivers’ native language, and audio recorded. A research assistant, who was also a native speaker of *Dholuo,* took notes during the groups, transcribed the audio recordings in their original language and translated them into English. An experienced professional speaker checked the accuracy of translation against the original *Dholuo* text. Corrected transcripts were imported into NVIVO software for management and analysis. Data were analysed using a thematic content analysis approach. The transcripts were read in detail and codes emerging from the text were identified. The transcripts were then coded according to the agreed coding scheme. The coded texts were subsequently discussed and the codes collapsed into agreed themes that represented the key elements of the perceptions and experiences described in the FGDs.

## Results

Thirty-four caregivers participated in four FGDs, two from each arm of the trial (Table 1). All participants were women between 20 and 66 years old. The majority, 20/34 (58.8%) were aged below 30 years.

**Table 1:** Characteristics of caregivers who participated in post-trial focus group discussions

FGD number	Trial arm	Number of participants	Age range (years)	Duration (min)
1	Intervention	9	20–48	40
2	Control	9	29–66	50
3	Intervention	8	20–30	45
4	Control	8	20–28	50

Four key themes relating to differences in experiences at health facilities during the trial and under routine conditions that had the potential to influence treatment adherence emerged from the data. These were: intervention and trial specific procedures; comprehensive implementation of national treatment guidelines; availability of medicines; and, patient/provider relationships and service efficiency.

### Trial specific effects

Although there was no difference in adherence between the intervention and control arms of the study, caregivers in the intervention group said the SMS reminders had been effective in reminding them to administer the medicine:

*First of all, it was the short messages that reminded you to give the child medicine…Anytime you were about to forget, the phone reminds you, the message comes… So it was good they used phones.*

[FGD 1]

The caregivers in the intervention arm also reported that the SMS reminders had helped them administer the medicine at the correct times as advised by the health workers:

*Especially the short messages that reminded you the time for giving medicine, that helped us a lot because sometimes you were going to forget, and they send you that message, so you give the child medicine without passing the time.*

[FGD 3]

While only the participants randomized to the intervention received the SMS text message reminders, all participants provided their consent to take part in the trial during their visit to the health facility with a sick child, received trial-specific instructions from the health workers about the need to keep empty blister packs, and were informed that they could expect to receive a home visit to check the packs. The data from the discussions suggest that these instructions and practices contributed to the high adherence among both intervention and control group patients. The caregivers in both arms reported that they tried hard to give all the doses because they knew a health worker was going to visit them to check the blister pack:

*…the messages were being sent and they themselves* [the trial health workers] *were also coming… I used to be lazy in giving medicine to the child, so they told me they would come after some days… they said that when they come the packet that holds the medicine must be there so I tried hard to give medicine until I finished.*

[FGD 1]

*…us as mothers, when we give the child medicine and he becomes a bit active, when there’s still more tablets, we stop administering and keep for the next child who will fall sick and the child doesn’t finish the dose. ….We don’t usually finish medicine, even when you go to homes you will find there’s medicine that someone took but didn’t finish the dose. But the study helped us that you have to finish the dose.*

[FGD 2]

The practice of not completing the full treatment dose was widely discussed and reported as being common before the trial in all groups. The emphasis in the trial (in both intervention and control arms) to complete all doses was said to have helped ensure that all the tablets were taken and this was perceived to have had a positive impact on the recurrence of malaria:

*There are some parents who only see when the child is sick and after giving the child medicine, he does not finish the dose, so the malaria will come back to the child. But on this side those doctors come until the medicine is finished. So they follow up until the medicine is finished, after the dose is finished it’s not easy for the malaria to come back.*

[FGD 3]

### Comprehensive implementation of national treatment guidelines

The conduct of a clinical trial requires that all participants receive ‘quality care’ and this usually involves the comprehensive implementation of national treatment guidelines. A striking feature of the discussions with caregivers about their experiences during the trial, compared to routine conditions, was the extent to which caregivers appeared unused to receiving malaria treatment as per the national treatment guidelines for dispensing and counselling on AL use (Box 1) and the impact that receiving such treatment had on their perceptions and reported behaviour.

#### Box 1 National dispensing and counselling standards for AL treatment [9]

1. Directly observe the first treatment dose at the health facility.
2. Show all caregivers of young children how to prepare the dispersible tablet prior to administration. Ensure he/she understands how to administer the same to the child prior to leaving the facility.
3. If vomiting occurs within 30 min after the drug administration, the dose should be repeated.
4. Emphasize that all 6 doses must be taken over 3 days even if the patient feels better after a few doses.
5. Directly observe the first treatment dose at the health facility.
6. If vomiting occurs within 30 min after the drug administration, the dose should be repeated.
7. Emphasize that all 6 doses must be taken over 3 days even if the patient feels better after a few doses.

### Experience of directly observed therapy

The Kenyan national guidelines state that the first dose of an anti-malarial drug should be given by directly observed therapy. During the discussions it emerged that none of the caregivers had experienced being observed by a health worker administering the first dose of AL at the health facility, or seeing how the dosing might be done:

*I have never seen a doctor* [health worker] *who gave my child medicine … I have never seen… there’s nothing that the study people did for us that these people have ever done for us, it’s different … I’ve never been given to start giving the child right here, that’s something that’s never happened, I’ve never seen.*

[FGD 2]

Observing the first dose being given and being instructed to follow the same procedures for subsequent doses encouraged the caregivers who had never seen the ‘doctor’ administer medicine to their children to follow the instructions given:

*If I’m to say why we followed the teaching, we followed the teaching like the doctor taught us on how to give the child medicine and when they are treating the child, they give him medicine there and then.*

[FGD 3]

In particular, this ‘teaching’ involved demonstration of how to prepare the AL dispersible tablet as well as the amount of water to put in a cup for dilution, messages that none of the caregivers had received in their encounters with the health facility prior to the trial:

*First they gave me something to measure the medicine with. I have to make sure and they even drew a line… I must make sure that I reach the line and they drew on the container that I would use to give the child medicine.*

[FGD 3]

### Advice if vomiting occurs

It was clear from the discussions that, under routine conditions, caregivers were not told how to manage if their child vomited after taking a dose of the drug:

*The difference that I see on this side of the study, they teach people how the child should be given medicine and the time to give the child medicine, if the child vomits, what to do but these people* [routine health workers] *don’t do that*.

[FGD 4]

In the trial context, all participants were told to administer the dose again if the child vomited within 30 min:

*They said, they said that if the child vomits the medicine, you should add him. You add him more medicine*.

[FGD 3]

The reasons for administering the drug again if the child vomits were also explained to the caregivers:

*And they told me…because when the child has malaria he gets nauseous and may vomit, you can try to avoid that. They told me when he vomits he should be given more medicine right there and then, the other medicine has gone to waste.*

[FGD 2]

The importance of administering AL after food to prevent vomiting was also explained to the caregivers:

*At the time my child was given medicine at the table, he vomited. So I was told that when I want to give him medicine he should have eaten before taking the medicine …What I have learnt here, when you want to give the child medicine, you have to make sure he’s eaten before it’s time to take medicine. That’s when the medicine will work well in his body.*

[FGD 2]

### Information on the dosing schedule

In discussions about the dosing schedule the participants spoke about the level of detail provided by the health workers during the trial and referenced a specific time as helping them to understand when they should administer the second and subsequent doses:

*So they were reminding us that after giving him medicine, you count eight hours then give him the second dose… so I counted with him* [the trial health worker] *to find out what time it will be after those eight hours… I was told after eight hours is when I should add him more medicine… so the fact that time was given I saw brought help.*

[FGD 2]

For some participants the mention of specific times between doses enhanced their ability to comply with the timing instructions:

*The difference is they* [health workers in routine care] *tell you to give him a spoonful in the morning and evening and the other guys* [trial health workers] *tell you the time to give medicine…I find it easy because the time that was said, 8:00.*

[FGD 1]

The fact that during the trial they wrote the dosing times on the medicine packet was also reported to be helpful in ensuring adherence but was not common practice under routine settings:

*They wrote for me the time I should give him the medicine, they wrote it on the paper found in the Coartem.*

[FGD 4]

However, while details of specific times and providing a written reminder of the times was clearly valuable for many of the participants, it was the amount of time spent explaining what the writing meant and why the timing was important that appeared to have the greatest effect on the participants’ willingness and ability to follow the dosing instructions.

*The doctor wrote the time you started with on the packet so that you count those hours and he wrote down all of them, the times to give the child medicine and so I just set an alarm… we counted together on the table and I was asked what time…where will the eight hours fall? Now the time it falls on is what was written on the medicine. And he asked me, “When time reaches will you know?” “Yes I will know.” “How will you know?” And I told him, “I have a watch I will check”.*

[FGD 2]

This was in contrast to experiences under routine conditions:

*The pharmacy guy will see the paper and write medicine for you, he will not tell you that, “This medicine should be given to the child like this, do this, do this or do this,” he will just write on the medicine how it should be given to the child, he will write the number of times and give you the medicine and you go home. Now you will have to use your head and see how the child should take medicine….And if you ask him, he will answer you harshly or he won’t answer you… he doesn’t have time, he doesn’t have time to sit down with you and explain to you that.*

[FGD 4]

As explained by one of the participants, the detailed explanations had also helped them to interpret subsequent dosing instructions commonly written on medicine packets:

*It helped me, even when my son was sick and I went to the hospital and was given medicine written 2* *×* *3 or 2* *×* *2 now I just know that if it’s 2* *×* *2, I should give him at this time and this time. If it’s 2* *×* *3 I also see what to do, I don’t skip a day or time.*

[FGD 1]

Furthermore, the participants said that during the trial the health workers not only explained to them how to administer the medicine but also undertook checks to ensure that they had understood the instructions:

*After they had given me the medicine they explained to me how to use it, when to give it and it shouldn’t pass…they will explain to you very well. Until they ask whether you’ve understood what they’ve explained.*

[FGD 3]

### Emphasis to complete all doses even if the child feels better after a few doses

Completing all doses of the medicine was discussed by the participants not just in terms of having an empty blister pack to show to the trial staff during follow-up visit at home but also in the context of the explanations that were given of its importance:

*After they had given me the medicine they explained to me how to use it, when to give it and it shouldn’t pass. They told me that and told me, “Ensure the child finishes the dose”. They said the child must finish all the dose, you give him.*

[FGD 3]

In particular, the information that they had been given on the importance to their child’s health in completing the full dose was reported by the caregivers to have had an impact on their behaviour:

*Since I entered KEMRI I ensure they finish the dose especially the malaria dose. If you give it as required and finish it, it’s hard for your child to fall sick with malaria again ……… In the past after I have given the child two and he becomes active, I stop but now I can see if you give it all you see a change, it takes time before you treat the child again. But if you give him two and stop, three days will go by and he becomes sick again. That’s why I can say I have changed.*

[FGD 3]

### Availability of medicines

A key difference between the care the participants reported receiving under routine conditions compared to the care they received while in the trial was the constant availability of the anti-malarial medicine, AL. The participants across all groups reported that during the trial the malaria medicine was constantly available:

*…when the study people were here malaria medicine was there but after they left there was only Panadol, for malaria.*

[FGD 1]

*They* [trial health workers] *made sure that the medicine that the child was supposed to receive, was given in full and there was no payment, they gave all of it to you.*

[FGD 3]

As this participant describes, when malaria medicine is not available at the health facility then they have to go and buy it from elsewhere:

*And if you bring to the other side* [routine care] *they will tell you some medicine is missing and you should go buy.*

[FGD 3]

The participant from FGD 1 explained that finding funds to cover the cost of the drug was difficult:

*There was no malaria medicine there and so they didn’t give her. I went outside to buy at 120… going to buy medicine was hard for me.*

[FGD 1]

### Relationships between caregivers and trial health workers and efficient service

A further and perhaps most commonly remarked upon difference between treatment in the trial compared to routine conditions was the interaction and relationship between caregivers and trial health workers. The caregivers said that they felt treated like “queens” by the trial health workers who accompanied them through the process, explaining how things work and taking responsibility for the health of the child:

*We were being treated as the queen let me say that, because when you arrive, they take you to the doctor. It could be your first time there and you don’t know how things work…they are the ones who take you to the lab … in fact if you have a child they will leave you seated and he will go bring you medicine and bring it where you are. So you look at how someone took the responsibility of your child in the hospital.*

[FGD 2]

Once enrolled, the caregivers were given the trial coordinator’s telephone number to call in case of any emergency. This served to strengthen the relationship between the caregiver and the trial health workers:

*…when the child that was enrolled in the study was sick they even gave me their phone numbers …It placed me in a good place…*

[FGD 1]

Caregivers described the trial health workers as people with a ‘heart’, and they described being treated well:

*The relationship between me and the doctor that was treating the child, I am grateful to him and…he received people well and he was happy with people…he wasn’t harsh with people that’s why I am grateful to him…The KEMRI people have a heart. They can help many people*.

[FGD 3]

The relationship and treatment by health workers were described as being in stark contrast to their experiences in routine settings where the ‘doctors’ were described as usually being harsh and inconsiderate:

*They pay you no mind. They are not welcoming to people like the study people were close to people…and if you ask him, he will answer you harshly or he won’t answer you.. you find the person there has pulled their mouth and they are harsh, they will start harassing you that even if you have a child you will say, “I’m afraid to go back to those people”.*

[FGD 4]

Furthermore, several of the participants reported that under routine conditions they were often not told the cause of their child’s symptoms but just given a prescription to take to the pharmacy. It was clear in the discussions that the participants appreciated the time that the trial staff took in explaining the diagnosis they had reached and describing the treatment that their child would receive:

*The study people would tell you very well that your child has malaria…the study people tell you that you have malaria but on the other side* [routine care settings] *you don’t hear about the malaria…when the results are back from the lab, you are given and you take it to the doctor. After the doctor reads it, he is not free to tell you that your child has been found with malaria, he prescribes you a drug that you’ll read only if you are clever… There’s no one who will come to you directly to tell you that your child has been found with malaria. You’ll just be given a paper to take to the window* [pharmacy].

[FGD 2]

The caregivers also reported that their children had been managed fast and efficiently during the trial:

*So when I came I felt that they dealt with me well because they helped me quickly because my child was in a bad state…*

[FGD 2]

*But when you people* [trial health workers] *came to the hospital everything was done in a short time. The child is treated in the required time. That’s the good thing I saw in you people.*

[FGD 3]

And was contrasted to routine settings where care was reported to be slow, with long waiting times.

*The difference I saw, when you arrive with the child and he’s found with severe malaria they give him medicine there and then but the other side there’s nothing like that. You will line up even if the child is groaning, or dying in your arms, you will reach the doctor after maybe your child has died.*

[FGD 4]

Overall the participants in the FGDs, whether in the control or intervention arms of the trial, described a significantly enhanced quality of care for their children while they were enrolled in the trial compared to routine conditions.

## Discussion

This qualitative, exploratory study was undertaken to understand the mechanisms driving high caregivers’ adherence to AL therapy in children under 5 years of age with uncomplicated malaria that were observed in both intervention and control arms of the SMS trial in Kenya. The results suggest that a range of factors, including trial-specific procedures (e.g., consenting and providing information about the study), the relationships between trial participants and trial staff, and trial requirements (e.g., ensuring the provision of quality care as per national guidelines, and maintaining adequate supplies of AL) all contributed to the increased adherence seen in both trial arms. These results are in line with the findings of two recently published systematic reviews of studies on anti-malaria treatment adherence [4, 5]. The authors of both reviews suggest that patient awareness of trial involvement and the nature of the involvement can influence adherence outcomes. The review conducted by Bruxvoort et al. [4] found that the nature of the interaction between the research team and participants was associated with different levels of adherence among study participants. In studies where informed consent was collected from the participants at the time of first receipt of the medicine (such as in this trial) higher adherence was generally observed than when consent was obtained on the first follow-up visit. In addition, having a positive malaria test and observation of the administration of the first dose of AL by the dispenser (as was the case in this trial) were also found to have a positive effect on subsequent adherence. The review by Banek et al. [5], focusing more specifically on ACT, reported similar findings with studies in which participants were blinded to subsequent follow-up, reporting lower adherence rates than those in which participants were aware that they would be followed up at a later date. In the Kenyan trial examined in this manuscript the caregivers were recruited at the time of receipt of the first dose of AL, were instructed to keep the blister pack and were informed that they could be visited at home. The findings from this follow-up study, exploring the experiences of caregivers involved in the trial, suggests that this process had a significant impact on subsequent caregiver behaviour.

Informed consent is an ethical and legal prerequisite for the conduct of research with human participants but the timing and nature of the consenting process can influence both the willingness to participate in a study and subsequent behaviour. Many studies have demonstrated that participant understanding of what they are consenting to is not always congruent with the purpose of the research, with therapeutic misconception being amongst the most common misunderstandings [14, 15]. This is likely to be particularly true in communities that have had a long association with a research institute and where that institute has a reputation for providing quality health care to trial participants [16, 17]. Consenting to participate in a trial involves the development of new types of social relationships in which participants feel part of a ‘trial community’ [18]. Being part of this community involves a range of benefits but is accompanied by obligations of reciprocity in which the trial participants may feel obliged to reciprocate for the trial benefits by adhering to trial instructions, and in the expectation of positive outcomes. The reports from the women in this qualitative trial follow-up study suggest that being enrolled in the trial, even in the control arm, gave the participants a sense that they were being treated differently and provided with a better quality of care, potentially contributing to a willingness to adhere to the instructions provided.

While the process of consenting and the instructions and information provided at enrolment may have been a contributing factor to the trial outcomes, the experiences reported by the caregivers of sick children also suggest that the effective implementation of the national malaria treatment guidelines played an important role in influencing the trial outcomes. Many studies of malaria management across a range of countries in sub-Saharan Africa have pointed to the problem of the quality of care provided in many public health facilities, and particularly to the suboptimal practices related to medicine dispensing and patient counselling [19–22]. In such settings, the ethical requirement to ensure that clinical trial participants are provided with ‘gold standard’ care, following national guidelines, often results in significant resource inputs to participating health facilities [23, 24]. Data from the current study, and several other trials, suggest that these inputs (e.g., increased levels of staff, better trained staff, provision of medicines) can result in trial participants (whether in the control or intervention arms) receiving a much higher standard of care than experienced under routine conditions, particularly with respect to the time spent explaining to patients or caregivers the reasons for a particular treatment and the steps required to maximize treatment effectiveness [25, 26]. The extent of these inputs has led some commentators to label trials as short-term complex health interventions in themselves [24, 27], influencing the provision and uptake of care and improving disease management among providers, patients and caregivers, including adherence to guideline implementation and dosing schedules [24, 27]. In this study, the participants particularly mentioned that the trial staff had provided them with an adequate explanation of the dosing schedule, shown them how to administer treatment, observed administration of the first dose, and explained the necessity of completing all doses; information that, according to the national guidelines, should routinely be provided. Health worker adherence to national guidelines, particularly to dispensing and counselling standards, has been shown to be associated with adherence to a full course of malaria treatment [28, 29] and it is likely that the enhanced counselling that the caregivers received while the trial was being implemented, provided them with new knowledge and influenced their dosing practices.

The consistent availability of AL during the trial was also noted by the study participants. Several studies have found that stock-outs of anti-malarial medicines are common in many countries in sub-Saharan Africa, including Kenya [30–32]. Medicine availability is a key driver of treatment-seeking behaviour [33, 34]. Stock-outs of medicines at public facilities can result in patients and caregivers seeking treatment from the private sector where financial constrains can lead to the purchase of incomplete treatment doses; or to caregivers stopping treatment when their child appears cured and saving the remaining doses for subsequent disease episodes [33, 34]. The results from this study suggest that caregivers altered their adherence behaviour in response to enhanced counselling but that the availability of medicines also played a role.

Overall the caregivers involved in this exploratory study reported that their experiences during the trial were considerably different to the care they received under routine conditions. These differences suggest that a complex range of factors including: better counselling; the practical demonstration of how to give the medicine; the longer time spent with the health care provider and the focus of the provider on ensuring that the caregiver understood the instructions; the perception that for the duration of the trial, AL would be available at the health facilities; as well as the trial specific effects; all had an influence on adherence. No one single factor can be identified as being the primary driver of the adherence rates observed during the trial; rather the results illustrate the range of contextual factors (social relationships, communication methods, structural support etc.) that are required for trial implementation but that in themselves influence participant behaviour. In future adherence trials, attention needs to be paid not only to how and when participants are consented into the trial and how adherence is defined and measured, but also to the existing context of routine health care provision. Account needs to be taken of how care provision within the trial might differ significantly from the care received in a routine setting and how this is likely to affect trial outcomes.

## Limitations

This was a qualitative exploratory study which, by design, had a small sample size. The FGDs were conducted 5 months after the trial had been completed by which stage the participants might have had some difficulty in recalling their experiences during the trial. It became clear during the FGDs that their experiences during the trial and subsequently were very different and there remains that possibility that these differences might have been exaggerated during the re-telling. However, the participants accurately described the trial processes and their reports of their treatment are a valid reflection of their memories of their experiences. A longitudinal qualitative approach in which the participants are followed at intervals during and after the trial would have produced more ‘real-time’ data and involving a greater number of participants might have uncovered different experiences. However, the consistency in reports among the range of participants suggest that the key features of being a trial participant are described in these data.

## Conclusions

Numerous interventions have been developed to enhance adherence to anti-malarial medication with studies undertaken to evaluate their impact. Variations in definitions and measurement of adherence as well as the effects of different enrolment, consenting and follow-up procedures are all widely recognized as contributing to inconsistencies seen in patterns of adherence and in the scarcity of robust and conclusive evidence of intervention effectiveness. The results of this study point to the range of factors influencing adherence behaviour and how the difference in quality of care provided within a trial context can act as an additional significant confounder. Trials designed to measure the impact of interventions on AL adherence need to take account of the potential differences in care quality in trial and routine settings and their potential impact on trial outcomes.
